# Antagonistic fatigue crack acceleration/deceleration phenomena in Ni-based superalloy 718 under hydrogen-supply

**DOI:** 10.1038/s41598-023-33761-4

**Published:** 2023-04-26

**Authors:** Osamu Takakuwa, Yuhei Ogawa, Ryunosuke Miyata

**Affiliations:** 1 Department of Mechanical Engineering, Kyushu University, 744 Motooka, Nishi-ku, Fukuoka, 819-0395 Japan; 2 National Institute for Materials Science (NIMS), 1-2-1 Sengen, Tsukuba, Ibaraki 319-119 Japan; 3 Graduate School of Engineering, Kyushu University, 744 Motooka, Nishi-ku, Fukuoka, 819-0395 Japan

**Keywords:** Mechanical properties, Metals and alloys

## Abstract

Mechanical properties of structural alloys, including Ni-based superalloy 718 (Alloy718), are degraded when hydrogen (H) is supplied: hydrogen embrittlement (HE). The presence of H notably deteriorates fatigue crack growth (FCG) property, which renders the growth rate much higher and shortens the lifetime of the components operating in the hydrogenating environment. Hence, the mechanisms behind such acceleration phenomenon in FCG should be understood comprehensively toward developing promising alloys resistant to hydrogen occlusion. In particular, Alloy718 has a meager resistance to HE, even regularly displaying superior mechanical and physical performances. Notwithstanding, the present study unveiled that the FCG acceleration by dissolved H in Alloy718 can be negligible. An abnormal deceleration of FCG can instead be pronounced by optimizing the metallurgical state, a hopeful prospect in Ni-based alloys applied to the hydrogenating environment.

## Introduction

Ni-based superalloy 718 (Alloy718) displays superior mechanical properties: high-temperature performance combined with excellent strength-ductility balance, thereby utilized with the view to having better durability even in harsh environments, e.g., hydrogenating environment, oil well pipes, and rocket engines. Metallic materials exposed to hydrogen (H) exhibit their degradation in mechanical properties, including ductility^1,2^, fracture toughness^3–6^, and fatigue crack growth^7,8^, etc., widely called “hydrogen embrittlement (HE)” reported firstly by Johnson^9^. In particular, Alloy718 is extremely susceptible to HE^10^ despite its notable superiorities. For the practical usages of Alloy718 in the severe hydrogenating environment, HE events, especially in the fatigue process, should be elucidated to devise a fabrication process to improve its resistance against HE.

Two types of ordered coherent precipitates^11–13^: γ′′ (Ni_3_Nb), meta-equilibrium phase with D0_22_ structure, and γ′ (Ni_3_(Al, Ti)), equilibrium phase with L1_2_ structure realize the high strength of Alloy718 associated with its high-temperature performance. Another precipitate, δ (Ni_3_Nb), equilibrium phase with D0_a_ structure with semi-coherency to the matrix, is also intentionally nucleated along grain boundaries (GBs)^14^. The presence of δ phases ensures fine-grained microstructure because they function as obstacles against grain growth during the hot-working and solution treatment. Otherwise, the grains grow much more prominently when the processes are carried out beyond the solvus temperature of the δ phase^15,16^. The metallurgical states of the fine-grained microstructure accompanied by δ phase and the coarse-grained microstructure without δ phase are designated as “δ-FG” and “CG”, respectively, in the present study.

In the hydrogenating environment, the fracture in the δ-FG state originates at the δ/γ-matrix interface at GBs^17,18^, in which H atoms segregating at the interface weakens the interatomic bonding, i.e., hydrogen-enhanced decohesion (HEDE)^19,20^. One can thus conclude that the presence/absence of the δ phase is one of the controlling factors determining the susceptibility to HE of Alloy718. Nevertheless, HE still occurs even in the CG state without δ phase at GBs, shifting the predominant fracture sites to {111} slip planes (SPs) or annealing twin boundaries (ATBs)^2,21–23^. It is also noteworthy that two fine strengthening precipitates, γ′′ and γ′, dramatically alter the intrinsic dislocation glide behavior, contributing to an increasing heterogeneity and localization of the slip deformation. According to some fundamental hypotheses, diffusible H impacts the motion and stability of lattice defects^24,25^, suppressing dislocations cross-slip, further enhancing strain localization^26,27^, and nucleating superabundant vacancies beyond the thermal equilibrium concentration on the SPs. Such defect accumulation potentially diminishes the structural integrity along the slip plane, facilitating the emergence of the characteristic HE fracture modes. The change in fracture modes leads to ductility loss under monotonic tensile loading. Recently, crack initiation at SPs in this alloy stemming from the presence of H has been unveiled by devised test programs involving prestraining and intermediate temperature changes. H-segregation at SPs via thermal fluctuation played an essential role in the crack initiation at SPs^2^, emphasizing that the H-related failure is insensitive to temperature and deformation rate as long as the lattice diffusion of H is active enough.

As previously reported by the authors, crack propagation of Alloy718 under the fatigue cycle is also drastically accelerated by the presence of H^7^. We have systematically investigated the H-assisted (HA) fatigue crack growth (FCG), HAFCG, of CG state at a relatively high-stress intensity under a controlled internal H content, i.e., 90 wt ppm, by exposing the material to high-pressure gaseous H at an elevated temperature. The FCG rate became faster as the stress intensity increased compared to that under the absence of H. Hence, the presence of H deteriorates mechanical properties not only under static tensile loading but also in dynamic cyclic loading, i.e., fatigue.

In contrast to these hitherto-known HE-sensitive nature, in the present study, we provide an unforeknown insight that the presence of H surprisingly decelerates the FCG of CG material at low-stress intensities. It stems from generating an intense crack closure via altering the crack path geometry, opposing to δ-FG, whose FCG was still accelerated even at the same stress intensity factor range. Concerning the fatigue life design, the FCG behavior at low-stress intensities is more crucial than at high-stress intensities. The fact purports that the resistance to HAFCG in Alloy718 can be improved by optimizing the metallurgical state.

## Results

### Macroscopic FCG properties and fracture surface morphologies

**Figure 1:**
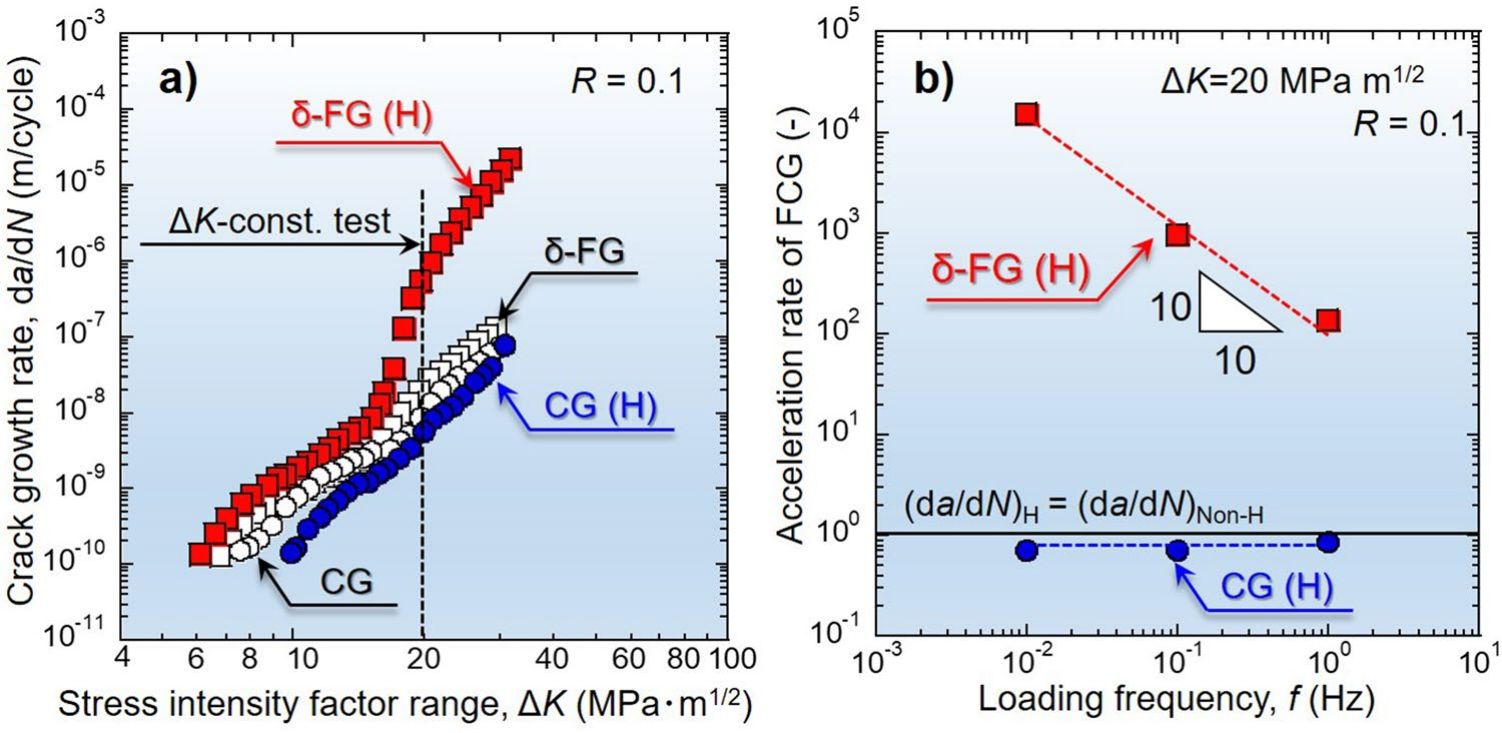
FCG properties under the absence and presence of H for δ-FG and CG: (**a**) Relationship between FCG rate, d*a*/d*N*, as a function of stress intensity factor range, Δ*K*, obtained by Δ*K*-increasing and -decreasing tests. (**b**) Relationship between acceleration rate of FCG, (d*a*/d*N*)_H_/(d*a*/d*N*)_Non-H_, as a function of loading frequency, *f*, obtained by Δ*K*-constant tests. (d*a*/d*N*)_H_/(d*a*/d*N*)_Non-H_ = 1 means no acceleration of FCG by the presence of H.

The FCG rate per fatigue cycle, d*a*/d*N*, for δ-FG and CG are plotted in Fig. 1a as a function of Δ*K* ranging from 6 to 30 MPa m^1/2^, combining the results acquired by the Δ*K*-increasing and -decreasing tests. Besides, Fig. 1b represents the relative acceleration rate of FCG, (d*a*/d*N*)_H_/(d*a*/d*N*)_Non-H_, as a function of loading frequency, *f*, at the Δ*K* kept constant at 20 MPa m^1/2^. The FCG rate in the H-charged δ-FG (marked as δ-FG(H)) represented an exponentially higher value than that for a non-charged state as Δ*K* elevated beyond 15 MPa m^1/2^, though the acceleration was not pronounced at Δ*K* < 15 MPa m^1/2^. As plotted in Fig. 1b, when the frequency was reduced an order of magnitude, the crack propagated ten times faster in δ-FG(H), exhibiting entirely time-dependent FCG. Namely, the FCG depends not on the applied load cycles but directly on the crack opening time.

However, the FCG behavior in CG(H) contrasted with δ-FG(H). Surprisingly, the presence of H decelerated the FCG rate at whole Δ*K* levels, in which the effect was more substantial near the threshold level of FCG with the d*a*/d*N* of an order of 10^–10^ m/cycle. Furthermore, the FCG rate in CG(H) was lower than that in the absence of H at any loading frequencies undertaken in this study (Fig. 1b). Thus, the crack propagated on a cycle-by-cycle basis, i.e., completely strain-dependent at any Δ*K* levels, even under the presence of H.

**Figure 2:**
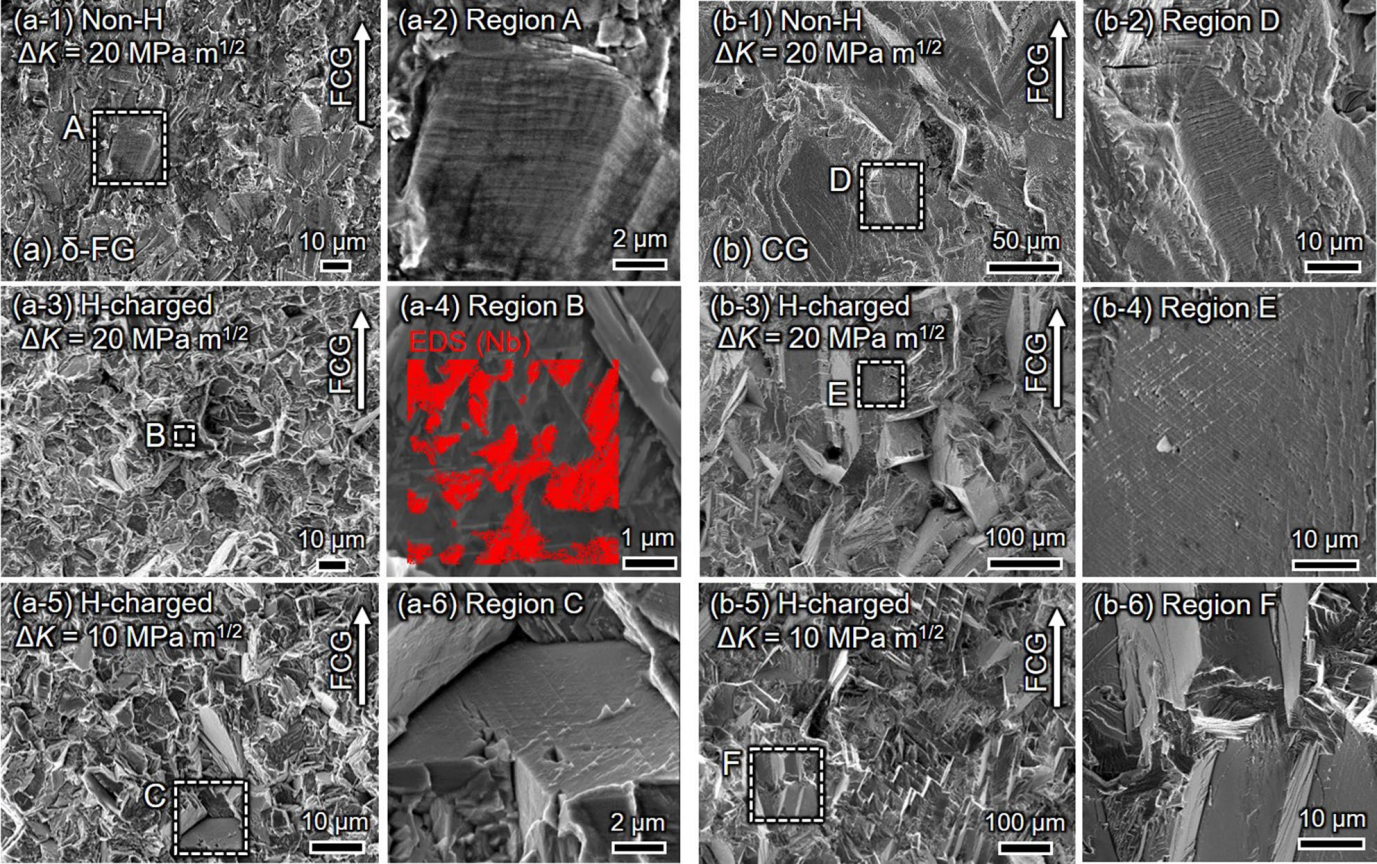
SEM images of the fracture surfaces for (**a**) δ-FG and (**b**) CG at Δ*K* = 20 MPa m^1/2^ and Δ*K* = 10 MPa m^1/2^: (**a-1,a-3,b-1,b-3**) are for the absence and presence of H, respectively, at Δ*K* = 20 MPa m^1/2^. (**a-5,b-5**) are for the presence of H in δ-FG and CG, respectively, at Δ*K* = 10 MPa m^1/2^.

**Figure 3:**
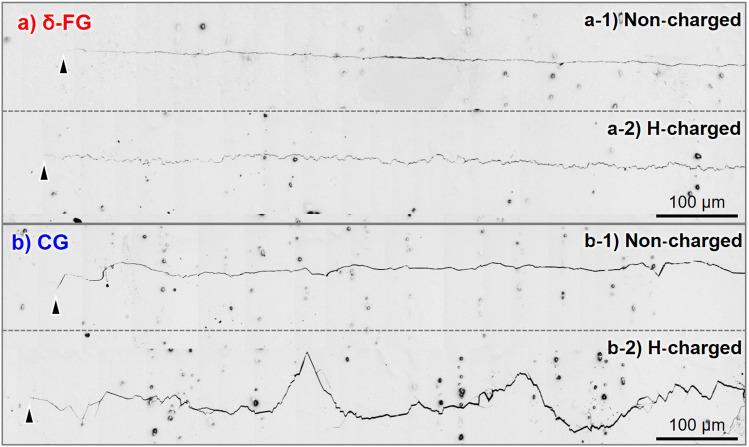
Macroscopic crack propagation pathways at Δ*K* = 20 MPa m^1/2^ in (**a**) δ-FG and (**b**) CG, obtained by optical microscope: (**a-1,b-1**) are for non-charged specimens. (**a-2,b-2**) are for H-charged specimens. The arrowheads denote the crack tips.

**Figure 4:**
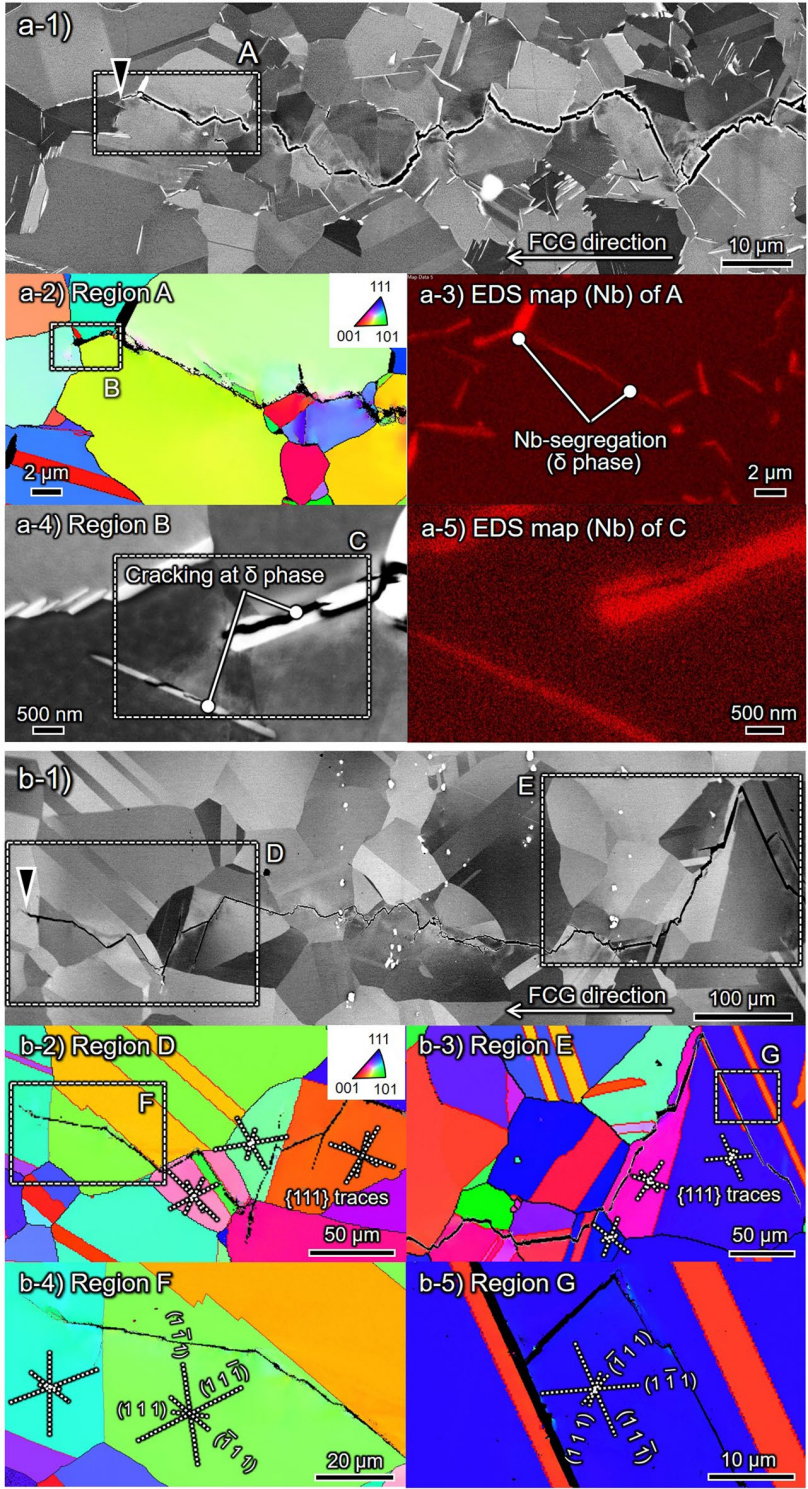
Crack path crystallography of (**a**) δ-FG and (**b**) CG in the presence of H subject to Δ*K* = 20 MPa m^1/2^: ECC images for overall aspects are in (**a-1,b-1**). Inverse pole figure (IPF) maps with respect to the loading axis at the regions A, D, and E are in (**a-2,b-2–b-5**) with {111} plane traces. The magnified ECC image of region B is shown in (**a-4**), besides corresponding EDS maps of Nb at the regions A and C are in (**a-3,a-4**), respectively.

**Figure 5:**
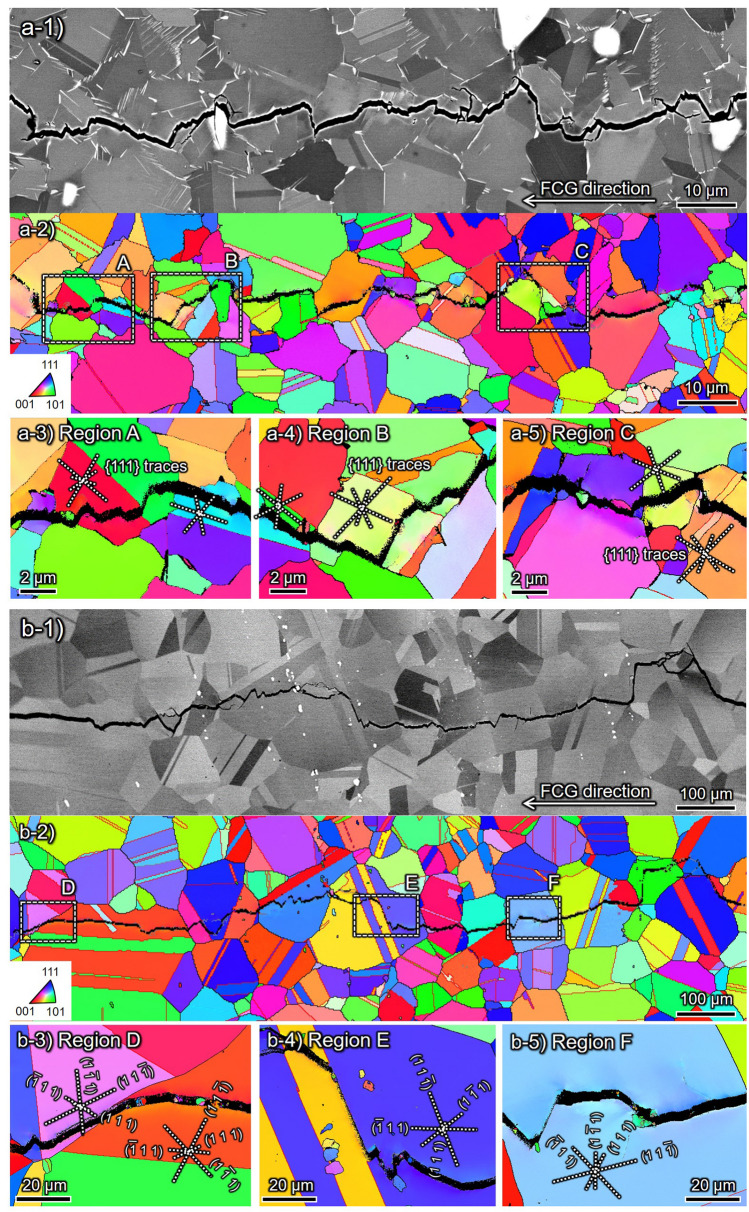
Crack path crystallography of (**a**) δ-FG and (**b**) CG in the presence of H at Δ*K* = 10 MPa m^1/2^: ECC images for overall aspects are in (**a-1**) and (**b-1**). Inverse pole figure (IPF) maps with respect to the loading axis at the regions A–C for δ-FG and D–F for CG are in (**a-3–a-5,b-3–b-5**) with {111} plane traces.

**Figure 6:**
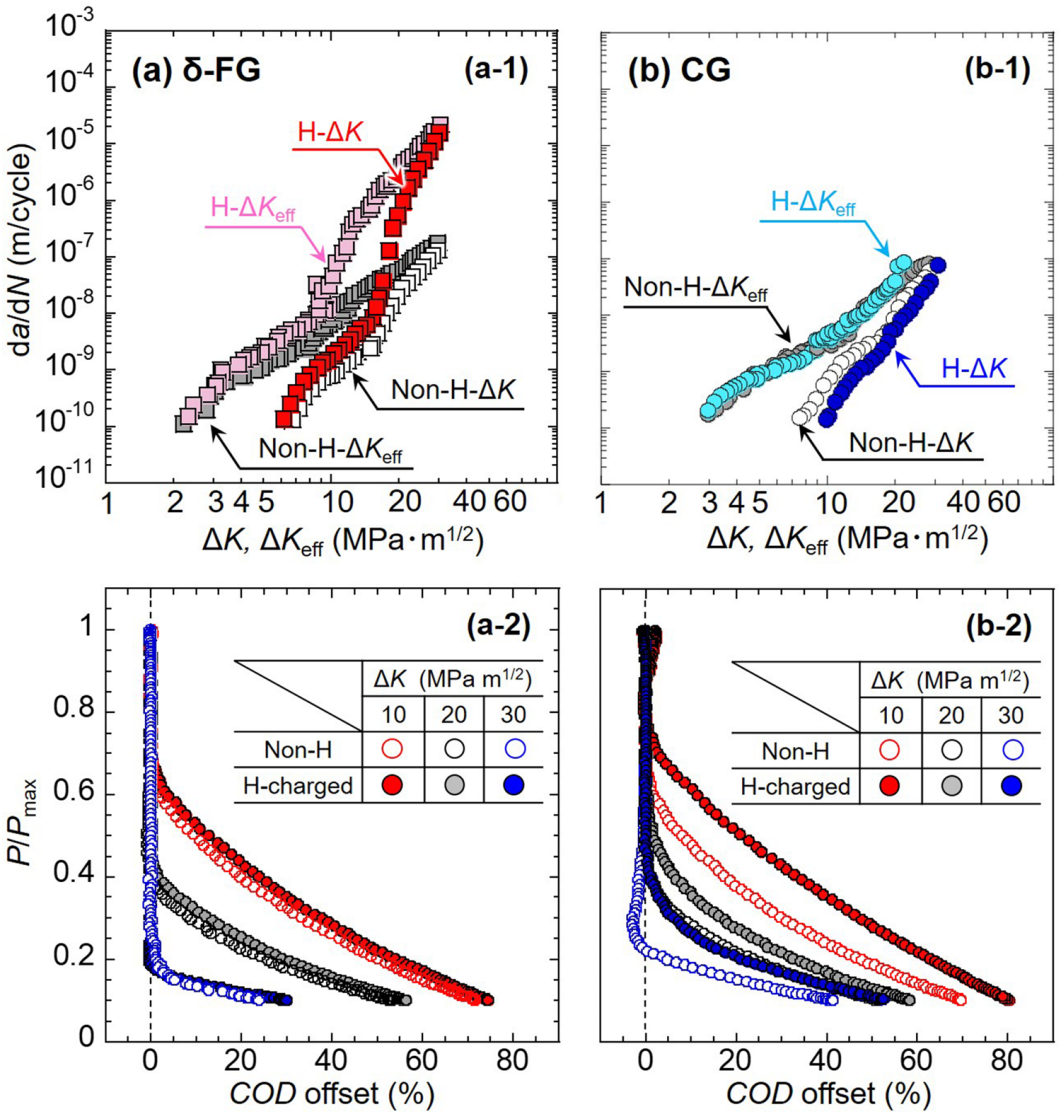
The FCG rate, d*a*/d*N*, – effective stress intensity factor range, Δ*K*_eff_, diagrams, together with the results of the crack closure analyses for (**a**) δ-FG and (**b**) CG. Δ*K*_eff_ is given by subtracting the stress intensity factor for crack closing, *K*_cl,_ from the maximum stress intensity factor, *K*_max_. The *K*_cl_ is provided by the closure analyses in the “Method” section. Crack closure propensity on the relationship between load and compliance offset by Eq. (1) at Δ*K* = 10, 20, and 30 MPa m^1/2^ for (**a-1**) δ-FG and (**b-1**) CG. Load is divided by its maximum value. Solid and hollow marks denote the datasets for H-charged and non-charged specimens, respectively.

Figure 2 shows the fracture surfaces of (a) δ-FG and (b) CG at Δ*K* = 20 MPa m^1/2^ and 10 MPa m^1/2^ in the absence and presence of H. As represented in Fig. 2a-2,b-2, ductile striations covered the fracture surface entirely in the H-absence in both δ-FG and CG. These striations are directed perpendicularly to the crack growth direction, reflecting the blunting and re-sharpening of the crack under loading and subsequent unloading processes per cycle. That is, the FCG under the absence of H was associated with intense local plasticity as given by the conventional slip-off model^28^, raising a well correspondence between the average interspacing of the striations and the macroscale FCG rate. Under the presence of H, however, the fracture surfaces thoroughly differed from the ductile striations, which exhibited brittle distinctions irrespective of the metallurgical states. In δ-FG, geometrical patterns decorated on the faceted fracture surface were proved to be Nb-rich δ phase by energy dispersive X-ray spectroscopy (EDS) mapping in Fig. 2a-4: the fatigue crack might propagate through the δ -precipitates decorating GBs. In CG, the fracture surface was covered by undulated zigzag patterns, as shown in Fig. 2b-3, consisting of large planar facets with multi-directional slip traces (cf. Fig. 2b-4). The dimensions of individual facets possessed an equivalent length scale with a grain size on the order of a hundred microns. When reducing Δ*K* from 20 to 10 MPa m^1/2^, the crack propagation morphology in δ-FG transitioned from the δ phase-related aspect to the planar faceted one (Fig. 2a-5,a-6), despite no Δ*K*-dependent change was confirmed in CG. The morphology transition indicates the presence of threshold stress intensity to encompass the δ phase-related GB cracking, which should be around Δ*K* ≈ 15 MPa m^1/2^ where the FCG acceleration became pronounced (Fig. 1a). The planar morphology with zigzag patterns in δ-FG has coincided with that exhibited in CG, except for the size of the asperities, i.e., ≈ 10 μm in δ-FG and ≈ 100 μm in CG, depending on each grain size. The significance of the asperities is directly related to the magnitude of crack deflection, as demonstrated in what follows: the larger the asperities, the more intense the crack deflection.

To visualize the H-effect on the crack deflection behavior in CG, the overall crack propagation paths captured by optical microscope are shown in Fig. 3, together with those in δ-FG, subjected to Δ*K*-constant tests at Δ*K* = 20 MPa m^1/2^. The crack deflection occurred most significantly in CG under the presence of H, corresponding to the large zigzag pattern on the fracture surface shown in Fig. 2b-3. Here, one can expect that such substantial crack deflection in H-charged CG invoked an intense roughness-induced crack closure (RICC). The occurrence of RICC potentially leads to the abnormal deceleration of FCG by the dissolved H.

The emergence of these distinct fracture morphologies in δ-FG and CG under the presence of H should result from each microstructure-dependent characteristic crack propagation behavior. In order to elaborate the detailed crack propagation paths, the electron channeling contrast (ECC) images, crystallographic orientation maps, and associated EDS maps (only for δ-FG to detect δ-precipitates) of the mid-thickness lateral sections around the fatigue cracks are shown separately with Δ*K* levels as follows:

### Crystallographic crack path in the presence of H at ΔK = 20 MPa m^1/2^

Figure 4 showcases the ECC images and corresponding inverse pole figure (IPF) maps for (a) δ-FG and (b) CG in the presence of H obtained by Δ*K*-constant tests under Δ*K* = 20 MPa m^1/2^. Note that the FCG rate represented substantial acceleration at Δ*K* = 20 MPa m^1/2^ in δ-FG yet contradictory deceleration in CG (cf. Fig. 1), wherein the difference between δ-FG and CG manifested in their crack propagation pathways.

The crack path changed entirely from transgranular to intergranular in the presence of H in δ -FG, discerned by comparing the crack path and Nb-rich regions (cf. Fig. 4a-2,a-3,a-4,a-5), i.e., the crack propagated via δ-decorated GBs. By focusing on the crack tip zone, the δ/matrix interfaces were debonded, or δ phases were fractured, as depicted in the magnified view in Fig. 4a-4. Thus, the crack propagated predominantly along δ-decorated GBs rather than the grain interior regions, which resulted in the fracture surface with Nb-rich characteristic patterns (Fig. 2a-4). Furthermore, tiny secondary cracks with the order of 100 nm were nucleated ahead of the primary crack tip. The coalescence of the primary and secondary cracks could encompass the time-dependent autocatalytic FCG during the crack opening process.

Conversely, CG exhibited a prominently different FCG path from δ-FG. As can be discerned by the plane trace analysis, the crack insistently propagated through ATBs or {111} SPs. The propensity that the ATBs or {111} SPs are preferential crack paths in the CG coincided with the results at high-stress intensities such as Δ*K* = 30 or 50 MPa m^1/2^, given by a previous study by the authors^7^. For instance, as exhibited in Fig. 4b-4, after propagating along (− 111) ATB, the crack left the boundary while propagating along (111) SP, passed over (− 111) SP in a step-like manner, and deflected, then propagated along (− 111) SP through (111) again. It can be inferred that the preferential SPs under FCG should have been selected according to the local stress state at the proximity of the crack tip. Furthermore, Fig. 4b-5 shows an identical aspect: the crack propagated at a relatively large deflected angle to the global FCG direction, adhering to (11-1) SP, then substantially changed its direction. The FCG events persisting at the characteristic propagation path, such as {111} SPs in CG, invoked the large asperities on the fracture surface (cf. Fig. 2b-3).

### Crystallographic crack paths in the presence of H at ΔK = 10 MPa m^1/2^

Figure 5 showcases the ECC images and corresponding IPF maps for (a) δ-FG and (b) CG, respectively, in the presence of H at Δ*K* = 10 MPa m^1/2^ obtained by Δ*K*-decreasing tests. The crack path did not depend on Δ*K* levels in the case of CG, propagating preferentially along {111} SPs or ATBs similar to the aspects at Δ*K* = 20 MPa m^1/2^. Notwithstanding, a notable finding was that the crack paths in δ -FG changed drastically from the intergranular (cf. Fig. 4a) to the transgranular site by decreasing Δ*K* from 20 to 10 MPa m^1/2^. The predominant crack pathways transitioned from the δ-decorated GBs to {111} SPs or ATBs as a similar trend to CG. Such an alteration also emerged in the fracture surface shown in Fig. 2a-5,b-5. The difference in the scaling of the asperities between δ-FG and CG stemmed from their grain sizes.

### Crack closure evolution in CG by the presence of H

Figure 6a-1,b-1 show the applied load normalized by its maximum value, *P*/*P*_max_, as a function of the crack-mouth opening displacement (*COD*) offset, defined in the “Method” section, for Δ*K* = 10, 20, and 30 MPa m^1/2^. The corresponding FCG rate, d*a*/d*N*, as a function of effective stress intensity factor range, Δ*K*_eff_, in the absence and presence of H for (a) δ-FG and (b) CG are depicted in Fig. 6a-2,b-2. In Fig. 6a-1,b-1, as the applied load decreased during the unloading process, the *COD* offset deviated from 0% at a certain load level, demonstrating the emergence of crack closure^29^, which depended on Δ*K*. At the lower Δ*K*, the crack closure is ensured at higher load levels, e.g., Δ*K* = 10 vs. 30 MPa m^1/2^. In general, plasticity-induced crack closure (PICC) is the leading cause of Mode I crack propagation associated with blunting/re-sharpening processes during the fatigue cycle^28,30^. For δ-FG, the curves of non-charged and H-charged states overlapped regardless of Δ*K* levels (Fig. 6a-1), which means that the magnitude of crack closure was not different between the absence and presence of H. In contrast, the curves of the H-charged CG diverged obviously from the H-absence for all Δ*K* levels (Fig. 6b-1). The fact indicates that the presence of H further augmented the crack closure in addition to the ordinary PICC. Referring to the FCG rate vs. Δ*K*_eff_ in Fig. 6a-2,b-2, all datasets transitioned toward smaller Δ*K* by subtracting the crack closure effect. Furthermore, the divergence of the FCG rate between the absence and presence of H was eliminated in CG (cf. Fig. 6b-2). Thus, the crack closure event induced by the presence of H rationalizes the abnormal deceleration of the FCG in CG. The theoretical approach to quantify the crack closure evolution from the perspective of RICC will be included in the “Discussion” section.

## Discussion

Overviewing the series of experimental insights in the present study, the extraordinary FCG behavior ascribed to the presence of H can principally be divided into two events: (i) the autocatalytic acceleration of FCG in δ-FG at Δ*K* > 15 MPa m^1/2^, and (ii) the deceleration in CG at all the Δ*K* levels. The crack insistently passed through δ-decorated GBs in the former (i), whereas it selected {111} SPs or ATBs in the latter (ii). This divergent behavior will be discussed separately in this section.

As shown in Fig. 1a, the FCG in δ-FG was substantially accelerated for Δ*K* > 15 MPa m^1/2^, which became more pronounced as the loading frequency, *f*, was decreased (cf. Fig. 1b). Note that the substantial acceleration of FCG stemmed from the crack propagation at the δ-decorated GBs, which left an Nb-rich geometrical pattern on the fracture surface (Fig. 2a-4). The presence of H induced the δ phase or δ/matrix-interface cracking (cf. Fig. 4a-4). The tiny secondary cracks ahead of the primary crack tip indicate that the intergranular cracking nucleated at the δ-decorated GBs in the region of the high-stress field at the proximity of the primary crack tip and coalesced with each other. Such a process leads to the autocatalytic unstable crack propagation in a time-dependent manner, i.e., not “strain-controlled” but “stress-controlled”. In other words, the phenomenon is not “dynamic”, but “static” crack growth, which is directly dependent on the “crack opening time”. Namely, the crack propagation continues during which the stress intensity at the crack tip exceeds the static crack growth resistance, i.e., the threshold stress intensity for H-induced cracking, *K*_IH_^4,31,32^. It has been reported that unstable time-dependent crack growth occurs in high-strength steels, e.g., martensitic steels^33,34^, associated with secondary cracks under the fatigue process in the presence of H^35^. The FCG acceleration event is synonymous with a manner caused during monotonic tensile loading.

Failure at δ-decorated GBs during tensile deformation has often been reported in the HE of Alloy718^17,18,36,37^. Tarzimoghadam et al.^18^ performed H-charging to Ni–Nb binary alloy with intentionally coarsened δ phase and verified the H segregation by combining multiple H-detection techniques. They concluded that H atoms segregate clearly at the δ/matrix interface, which is responsible for the cracking at the interface. The H segregation at the incoherent precipitate/matrix interfaces and resultant fracture has been reported not only in Ni-alloys but also in other alloys, e.g., Al–Zn–Mg alloys^38^. It can be speculated that the H atoms trapped at the δ/matrix interface reduce interatomic bonding force, inducing premature failure following the HEDE model^19,20^.

Based on the local equilibrium theory proposed by Oriani^39^, we can estimate the trap site occupancy of H, ${\theta }_{T}$:

1 $$\frac{{\theta }_{T}}{1-{\theta }_{T}}=\frac{{\theta }_{L}}{1-{\theta }_{L}}\text{exp}\left(\frac{{E}_{b}}{RT}\right),$$

where ${\theta }_{L}$ denotes lattice site occupancy, which comes from the H content in bulk. *E*_b_ presents the binding energy of the trap site, i.e., δ-decorated GBs, with H atoms. *R* and *T* are universal gas constants and temperature, respectively. In the present study, ${\theta }_{L}$ can be estimated to be 5.60 × 10^–3^ from the H content of 96 wt ppm that preferentially dissolves into octahedral sites in face-centered cubic (FCC) crystal. *E*_b_ is 30 kJ/mol for the δ/matrix interface, as reported by Turnbull^40^. At *T* = 543 K (i.e., H-charging temperature), the ${\theta }_{T}$ equals 0.81, whereas it asymptotically approaches 1 around ambient temperature. Thus, the δ/matrix interface can preferentially trap H, as already revealed experimentally. These trapped H trigger intergranular cracking during the crack opening process beyond the threshold level, *K*_IH_.

From the rapid changes in the trend of FCG rate and failure mode around Δ*K* ≈ 15 MPa m^1/2^, it can be inferred that the stress intensity acting on the GBs at the crack tip proximity did not reach a sufficient level to crack the δ/matrix interface or δ phase itself at the low Δ*K* domain (Δ*K* < 15 MPa m^1/2^). The acceleration of FCG with δ phase-related intergranular failure was thereby infeasible, urging the crack to instead pass through {111} SPs or ATBs similarly to CG (cf. Fig. 5b). Namely, one can directly link the resistance to static crack growth in the presence of H to the fracture criteria of the H-segregated δ/matrix interface or δ phase interior. An accurate evaluation of such specific resistance, which seems to depend on the H content and temperature, should be a vital piece for the practical usage of Alloy718 in hydrogenating environments.

In the case of CG, H decelerated the FCG rather than accelerating it (cf. Fig. 1), a trend absolutely differed from that in δ-FG. The lower the Δ*K*, the more pronounced the deceleration became. As shown in Fig. 2b-2,b-3,b-5, the relevant fracture surface with substantial asperities and undulated crack pathways suggests that the failure mode was latently changed by the presence of H, even though the FCG acceleration was not detected in Fig. 1. The plane trace analyses on the EBSD image of the crack path in Fig. 4b-4 indicated that the crack propagated through ATBs and some preferential {111} SPs. This tendency was consistent with what the authors reported in our previous study focusing on a higher Δ*K* regime (30 ≤ Δ*K* ≤ 50 MPa m^1/2^). It has been represented that, as Δ*K* increased, the FCG acceleration became more pronounced, even with no acceleration below Δ*K* ≈ 30 MPa m^1/2^. At a higher Δ*K* regime, e.g., Δ*K* ≈ 50 MPa m^1/2^, time-dependent “static” crack growth emerged similarly to δ-FG in the present study by which the secondary cracks originated at the intersections of dislocation slip bands (DSBs) autocatalytically coalesced with the primary crack^7^. It is quite likely that the maximum stress intensity was not beyond the aforementioned resistance to the H-assisted cracking (*K*_IH_) at Δ*K* < 30 MPa m^1/2^ (*K*_max_ < 33 MPa m^1/2^ for *R* = 0.1) in the present study, then the FCG event exhibited “cycle-by-cycle” and “strain-controlled” crack propagation.

The ATBs and {111} SPs have already been reported to function as the initiation sites of H-related damage in the absence of δ-decorated GBs^21,37^. Nevertheless, if the ATBs cracking occurred in a brittle manner similar to intergranular fracture, the FCG rate would be on the order of 100 μm. That is, the ATBs have an equivalent length scale with the coarse grains in CG, which means that if an ATB with ≈ 100 μm was suddenly fractured in a completely brittle aspect, it should have appeared on the FCG rate with the order of 10^–4^ m/cycle. However, the change in fracture mode did not accelerate the FCG but rather slowed it down. It can thus be inferred that the FCG in CG was caused by the mere change in fracture mode due to the presence of H, i.e., from pure Mode I to local Mode II. Dislocations were emitted from the crack tip onto the SPs, and the resultant plane sliding propelled the crack tip forward. Equivalently, the ATBs cracking can also be ascribed to the framework of such Mode II crack growth since the ATBs with Σ3 character in FCC crystal consists of one of the four {111} planes where dislocations motion is available^41^. Furthermore, Zhang et al.^23^ reported that γ′′ tend to precipitate and coarsen at the ATBs, facilitating the deformation-induced fracture at the {111} SPs in the Nb-depleted precipitation-free zones (PFZ) generated in the extreme proximity of the boundary. Hence, the crack propagation along ATBs did not exhibit a brittle manner like the intergranular cracking such as δ-FG under Δ*K* > 15 MPa m^1/2^. In addition, the crack propagation in δ-FG with the presence of H exhibited the transgranular cracking through the ATBs and {111} SPs at low Δ*K* regime (Δ*K* < 15 MPa m^1/2^) like CG. The propensity indicates that the fracture modes at the low Δ*K* regime in δ-FG and CG are intrinsically identical.

The presence of H increased the crack opening load, decreasing the effective stress intensity factor range, Δ*K*_eff_, in the case of CG (cf. Fig. 6b-1,b-2). The concomitant crack closure evolution could trigger extraordinary FCG deceleration. Even though the crack deflection seems to render the actual crack pathway longer by referring to Fig. 3, the d*a*/d*N* − Δ*K*_eff_ curve of the CG in the presence of H coincided with the absence of one in Fig. 6. Hence, it is plausible that the reduction of the FCG in the CG by the presence of H stemed not from the change in the actual crack length per fatigue cycle but from the crack closure induced by the asperities on the fracture surface (RICC). Although the FCG associated with the crack tip opening/re-sharpening, i.e., Mode I, is generally affected by PICC, shear mode crack propagation, i.e., Mode II, was more favored locally at the crack tip zone with the aid of H dissolution, leaving the faceted surfaces and asperities on the crack-passed regions. The asperities on the fracture surface might trigger RICC owing to the contacting between the upper and lower faces of the deflected crack, whose wavelength can be a direct function of the grain size. From the geometrical profiles of the crack propagation path (cf. Fig. 3), the averaged crack deflection angle, *θ*m, was calculated by dividing the profile along the direction of global FCG at the interval of 5 μm. The stress intensity at which the premature crack face contact by the roughness, *K*_cl_, can be estimated by the angle using Eq. (2)^42,43^, followed by the derivation of Δ*K*_eff_ by Eq. (3):

2 $$\frac{{K}_{\text{cl}} \, }{{ K}_{\text{max}}}=\sqrt{\frac{k\text{tan}{\theta }_{\text{m}}}{1+k\text{tan}{\theta }_{\text{m}}}},$$

$$\left(k=\frac{3}{2}\frac{{ R}_{\text{s}}-1}{{ R}_{\text{s}}+1}, { R}_{\text{s}}=1/\text{cos}{\theta }_{\text{m}}, {\theta }_{\text{m}}=\frac{1}{n}\sum \limits_{i=1}^{n}{\theta }_{i} \left({\theta }_{i}={\text{tan}}^{-1}\frac{{Y}_{i}}{{X}_{i}}\right)\right),$$

3 $${\Delta K}_{\text{eff}}={ K}_{\text{max}}\left(1-\frac{{ K}_{\text{cl}} \, }{{ K}_{\text{max}}}\right).$$

The results showed that, at Δ*K* = 20 MPa m^1/2^, Δ*K*_eff_ were 20.0, 19.2 MPa m^1/2^ for δ-FG and 19.7, 15.9 MPa m^1/2^ for CG in the absence and presence of H, respectively. That is, the RICC reduced Δ*K* by about 4 for CG with the influence of H, which is in good agreement with the value separately calculated from the crack opening load in Fig. 6a-1,b-1. Note that the calculation merely focused on the contribution of RICC to the crack closure evolution, and the crack closure in other cases of δ-FG and CG in the absence of H might be derived from the ordinary PICC. The lack of significant RICC in δ-FG with the presence of H can be attributed to its grain size with an order of magnitude smaller than that in CG. Thus, unlike CG, the H-induced deceleration phenomenon did not appear despite the apparent operation of ATBs and SPs fracture.

From the viewpoint of the difference in grain size between δ-FG and CG, it has been found that the smaller the grain, the lower the susceptibility to H-related failure, especially in fcc alloys^44–46^. It is because the grain refinement can reduce microstructural stress/strain concentration at interfaces such as GBs and ATBs, which are the potential sites for damage accumulation. In the present study, CG rendered excellent FCG properties, and δ-FG showed autocatalytic FCG acceleration opposite to the insight reported by the past articles. The δ phase at GBs under the presence of H triggered the intergranular cracking (cf. Fig. 4), overwhelming the positive effect of the grain refinement. Alternatively, CG generated the intense RICC, reducing the effective stress intensity factor under cyclic loading.

**Figure 7:**
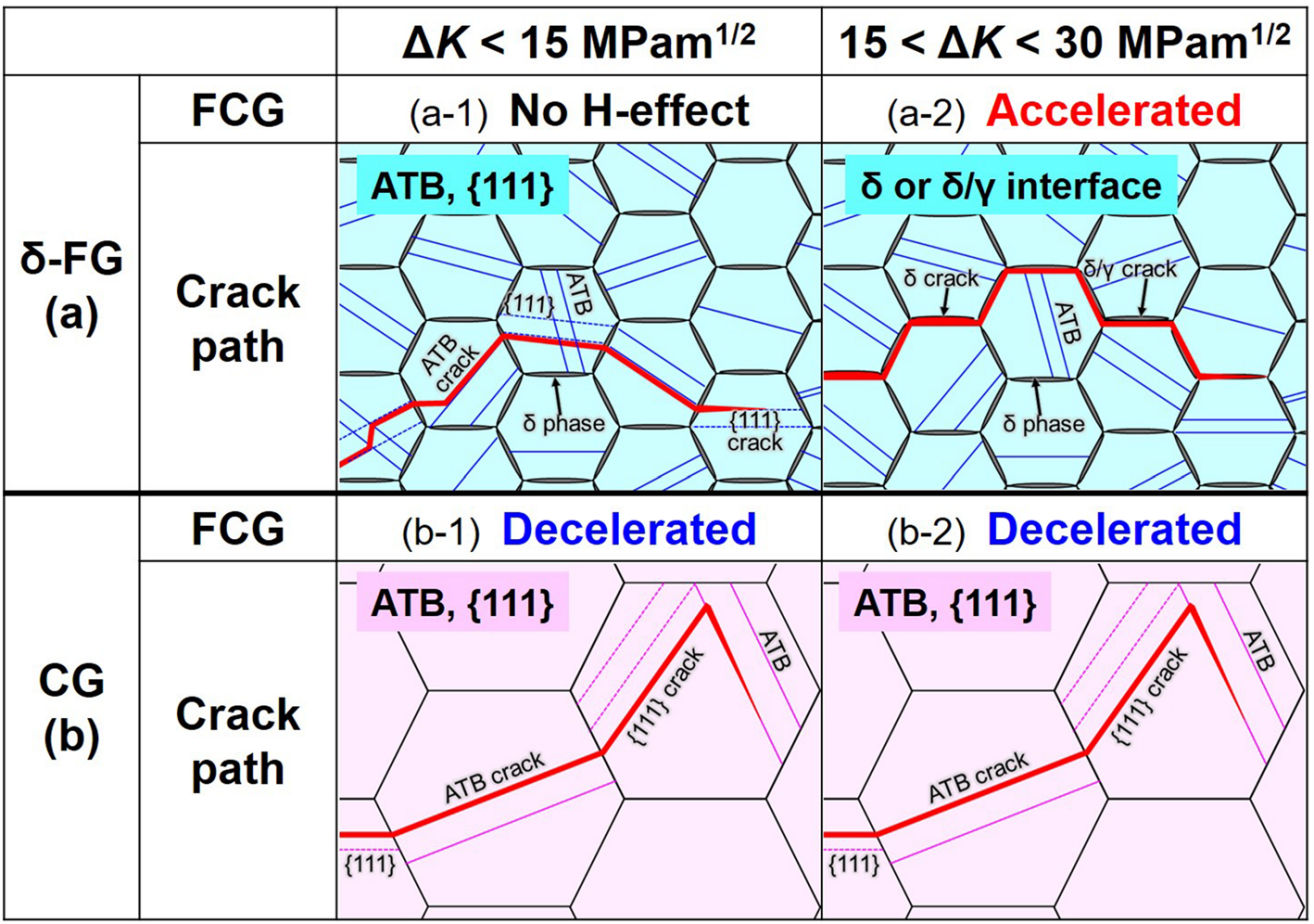
Schematic illustrations of the H-accelerated/decelerated fatigue crack growth (FCG): (**a**) stress intensity factor range Δ*K* < 15 MPa m^1/2^ for (**a-1**) δ-FG associated with transgranular cracking exhibiting no acceleration of FCG and for (**a-2**) CG associated with transgranular cracking exhibiting deceleration of FCG. (**b**) Δ*K* > 15 MPa m^1/2^ for (**b-1**) δ-FG associated with intergranular cracking along δ-decorated grain boundaries exhibiting substantial time-dependent acceleration of FCG and (**b-2**) CG associated with transgranular cracking exhibiting deceleration of FCG.

Figure 7 depicts a summary of the FCG-acceleration/deceleration events induced by the dissolved H in δ-FG and CG, classified into several types based on the Δ*K* levels. At Δ*K* < 15 MPa m^1/2^, no acceleration of FCG was detected for both δ-FG and CG. Instead, a deceleration was observed in the latter. The crack propagated mainly along {111} SPs or ATBs interior grain regions. Meanwhile, at Δ*K* > 15 MPa m^1/2^, the autocatalytic FCG acceleration occurred in δ-FG with time-dependent unstable crack growth, wherein a tenfold increase in crack opening time resulted in an order of magnitude increase in the crack growth rate. In this case, the cracks preferentially propagated along the GBs, i.e., the interfaces between grain boundary precipitates (δ phase) and the matrix phase. In contrast to δ-FG, CG did not exhibit the FCG acceleration at all, even at Δ*K* > 15 MPa m^1/2^. The crack propagation pathway was not affected by Δ*K* levels and was either {111} SPs or ATBs, which were responsible for the substantial crack deflection. Ultimately, the crack deflection invoked the intense RICC that directly reduced theΔ*K*_eff_, triggering an abnormal FCG deceleration in CG with the presence of H.

## Methods

### Material and specimen

The material examined was a Ni-based superalloy 718 (UNS-N07718) with 40 mm diameter fabricated by hot-drawing. The chemical composition of the alloy was 53.5Ni–18.5Cr–17.6Fe–5.0Nb–2.9Mo–0.94Ti–0.5Al in mass %. The solution annealing was done at 955 °C for 1 h for fine-grained microstructure with grain boundary precipitate δ phase (i.e., δ-FG), and at 1065 °C for 1 h for coarse-grained microstructure without δ phase (i.e., CG). Then, double-aging was performed at 721 °C and 618 °C for 8 h each for δ-FG, while at 760 °C and 650 °C for 10 h each for CG with subsequent cooling in Ar gas. The 0.2% proof stress and ultimate tensile strength for δ-FG were 1224 and 1450 MPa, whereas they were 1224 and 1390 MPa for CG.

Compact-tension (CT) specimens for the FCG tests were extracted so that the fatigue crack propagates normal to the drawing direction. The specimens were machined into a width of 33 mm and a thickness of 6.0 mm.

### Microstructures

**Figure 8:**
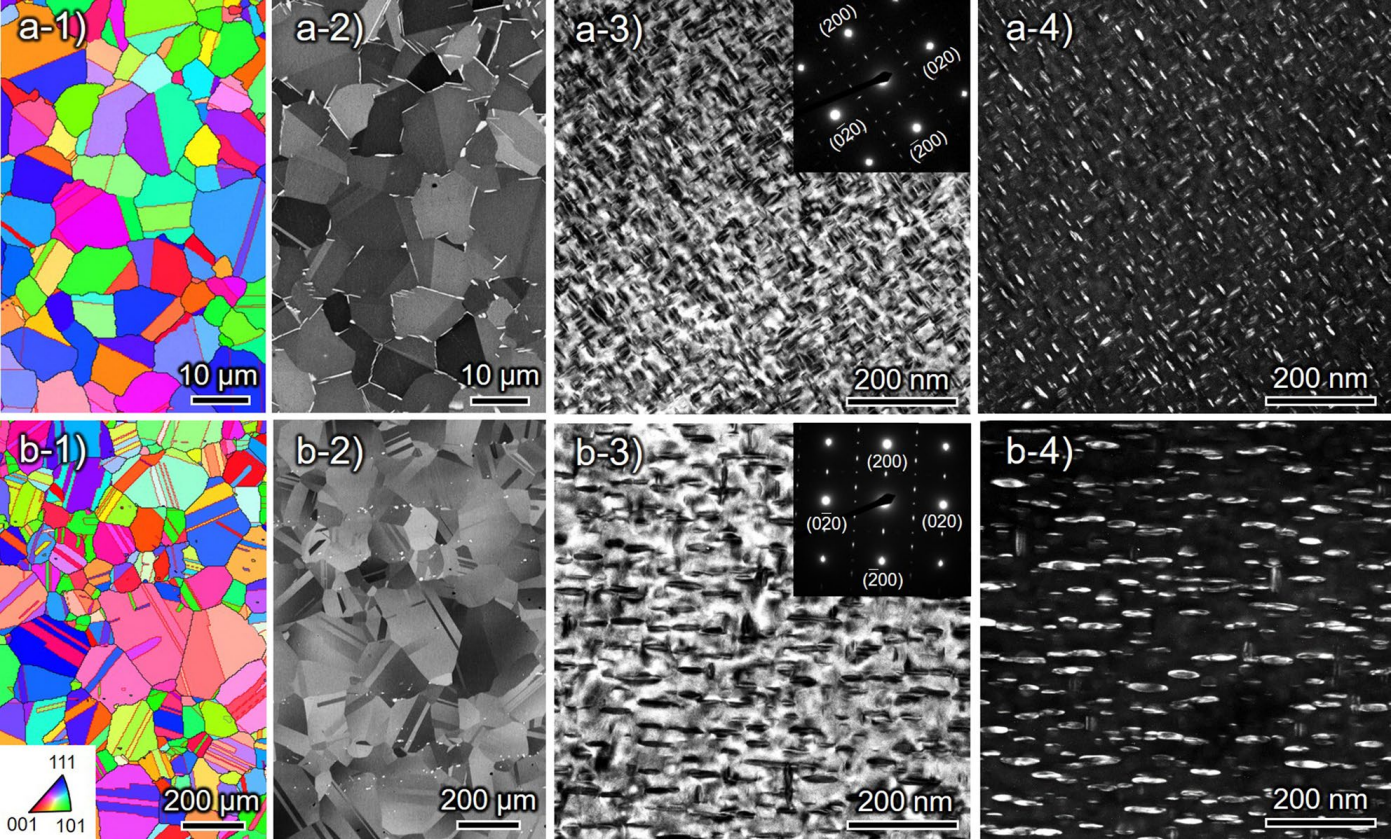
Microstrcutres observed by EBSD, ECC imaging, and TEM for (**a**) δ-FG and (**b**) CG: (**a-1,b-1**) Inverse pole figure maps with respect to the longitudinal direction; (**a-2,b-2**) ECC images; (**a-2,b-2**) Bright-field and (**a-3,b-3**) dark-field micrographs obtained from [001] zone axis.

The IPF maps obtained by electron backscattered diffraction (EBSD) and ECC images of the initial microstructures in δ-FG and CG are shown in Fig. 8a-1,a-2,b-1,b-2. The average grain size was approximately 8 μm for δ-FG and 130 μm for CG. The δ-FG contained δ (Ni_3_Nb: D0_a_) phases at GBs, which prevented grain growth during solution treatment and realized finer grains than CG. The δ phase did not generate in the CG as solution treatment was done beyond its solvus temperature. Figure 8a-3,a-4,b-3,b-4 show the bright-field and dark-field images obtained by transmission electron microscope (TEM) for δ-FG and CG displaying homogeneously distributed fine intragranular precipitates γ′′ (Ni_3_Nb: D0_22_) and γ′ (Ni_3_(Al, Ti): L1_2_), which are coherent with the matrix. The γ′′ precipitates play a primary role in strengthening the alloy, possessing a disc-like shape lying parallel to {100} planes of the matrix. The size of the precipitates was approximately up to 100 nm, and δ-FG had finer precipitates than CG, which stems from the slight difference in the aging temperatures.

### Thermal H-charging under a high-pressure gaseous environment

The CT specimens were thermally pre-charged by exposure to gaseous H under 100 MPa at 573 K for 200–300 h. This pre-charged condition enables to achieve uniform H distribution through the specimen thickness, which can be estimated by diffusion coefficient, *D*, temperature, *T*, and duration time, *t*, as $\sqrt {Dt}$. When $\sqrt {Dt}$ is beyond the half-thickness of the specimen, H should be uniformly distributed through the specimen. Accordingly, a longer than 170 h duration is enough when *D* = 1.50 × 10^–11^ m^2^/s at 573 K^47^. The H content was evaluated by gas chromatography-type thermal desorption spectroscopy with a heating rate of 100 °C/h. The total H content of δ-FG and CG were 96 and 98 wt. ppm, respectively. The desorption profiles are given in Supplementary Fig. 1.

### Fatigue crack growth (FCG) tests

Before the FCG tests, a pre-crack was introduced from the starter notch in each specimen by applying cyclic loading with Δ*K* of 15 MPa m^1/2^, *R* of 0.1, and *f* of 10 Hz. The crack length was continuously measured by the unloading elastic compliance method via a clip-on gauge attached to the crack mouth of the specimen. Two Δ*K*-changing FCG tests were carried out according to the ASTM-E647^48^ to investigate extensive FCG properties where Δ*K* ranging from its threshold level: Δ*K*-decreasing test for Δ*K* < 20 MPa m^1/2^ and Δ*K*-increasing test for Δ*K* > 20 MPa m^1/2^. The Δ*K*-increasing test was controlled so that a load range, Δ*P*, was kept constant, and thereby Δ*K* increased spontaneously as the crack grew. Meanwhile, in the Δ*K*-decreasing test, Δ*P* was successively decreased as the crack grew with a decreasing rate of 2 MPa m^1/2^/mm. The decreasing rate was carefully selected since the FCG rate is affected by a history of plasticity ahead of the crack tip. Additionally, the Δ*K*-constant test was also performed at Δ*K* = 20 MPa m^1/2^, changing with test frequency, *f*, as *f* = 0.01, 0.1, 1 Hz. It can be found whether FCG is stable, i.e., strain-dependent, or not under a fixed mechanical condition. When the FCG rate becomes higher at the lower test frequency, the FCG has not only strain-dependency but also time-dependency similar to the conventional delayed fracture. As acquired in the author’s previous work, H-charged Alloy718 (CG) exhibited time-dependent FCG at the high-stress intensity at Δ*K* of 50 MPa m^1/2^^7^.

### Electron microscopy characterizations

The initial microstructures were observed using EBSD analysis on the plane perpendicular to a longitudinal axis of the bar implemented by a JEOL JSM-IT800 field-emission scanning electron microscope (FE-SEM) operated at 15 kV. For characterizing the precipitation phases (γ′′ and γ′), a JEOL JEM-2100EM transmission electron microscope was employed with an acceleration voltage of 200 kV.

After the FCG tests, the specimens were halved along their mid-thickness regions. The cross-sections of one-half were carefully finished by buffing with a colloidal SiO_2_. We also characterized the crack cross-sections using EBSD and ECC imaging techniques by a JEOL JSM-IT800 to facilitate the visualization of underlying crack paths.

### Crack closure analyses

Crack closure phenomena, first noted experimentally by Elber^29^, emerges during fatigue crack advance within the loading/unloading process derived from the plastically-strained crack wake, i.e., PICC, as well as from the asperities on the fracture surface, i.e., RICC. While the crack faces contact each other (the crack is physically closed), the crack cannot propagate. In this situation, the stress intensity range at the crack tip is lowered than the nominally calculated value (Δ*K* = *K*_max_ − *K*_min_). The effective value, Δ*K*_eff_, can be estimated as (Δ*K*_eff_ = *K*_max_ − *K*_cl_), where *K*_cl_ denotes the stress intensity to close the crack. The closure event directly contributes to the FCG rate, especially at low-stress intensities, making FCG slower even though the nominal value (Δ*K*) is the same.

The closure level can be quantitatively evaluated via applied load, *P*, and crack mouth opening displacement, *COD*, relationship. When the closure emerges, the *P*-*COD* relation is deflected from the original linear response, and the divergence from the linearity becomes more prominent as the applied load gets lower less than the crack opening load, *P*_op_. The divergence given by the following equation is defined as the *COD* offset:

4 $$COD.offset={COD}_{\text{exp}}-COD/{COD}_{\text{exp}}\times 100 (\%),$$

where *COD*_ext_ denotes the load on the extrapolation line given by *P*-*COD* relation at the region where the crack is completely opening. We defined the *P*_op_ as the load with a *COD* offset exceeding 4%.

## Supplementary Information

Supplementary Figure 1.

## Data Availability

The datasets used and/or analysed during the current study are available from the corresponding author on reasonable request.
