# Supplementary figures and images for: Antagonistic fatigue crack acceleration/deceleration phenomena in Ni-based superalloy 718 under hydrogen-supply
Source: Sci Rep. 2023 Apr 26;13:6804. doi: 10.1038/s41598-023-33761-4 (PMC10133311; doi:10.1038/s41598-023-33761-4)

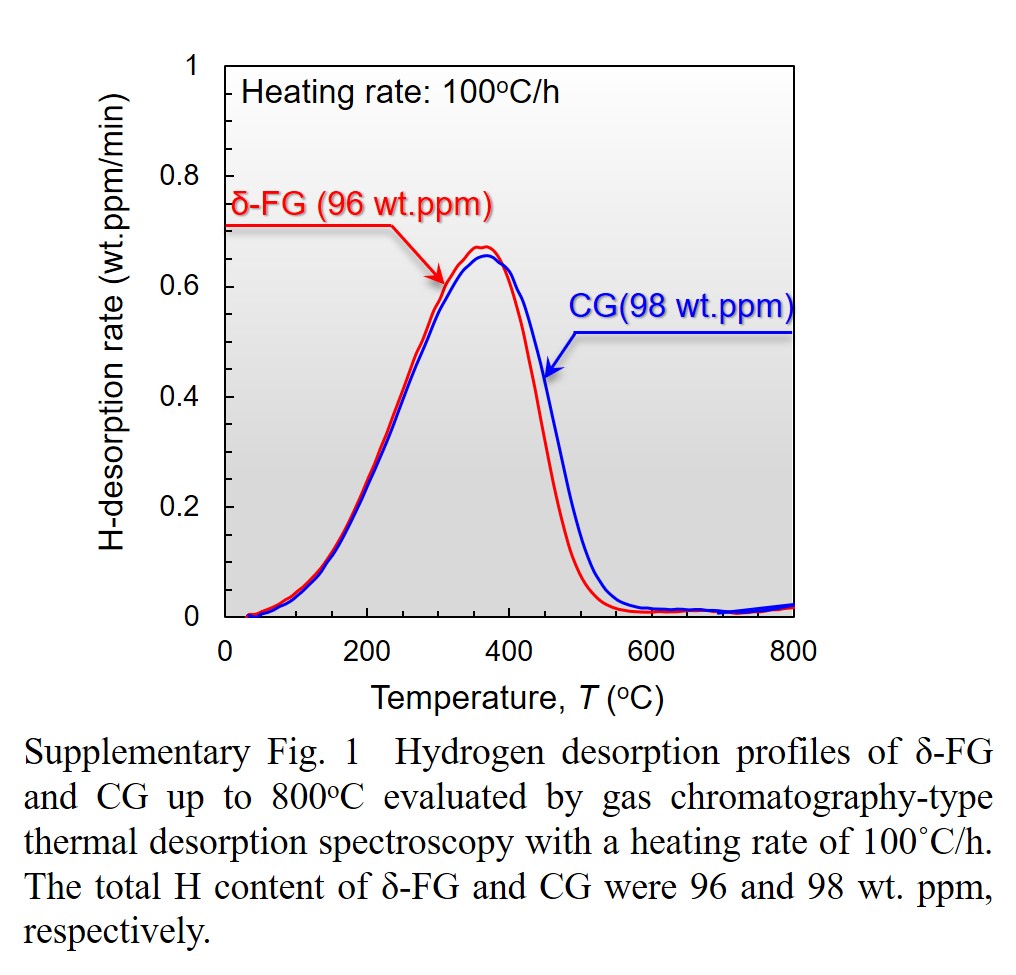

Supplement: Supplementary file 1 — Supplementary Figure 1. [file 41598_2023_33761_MOESM1_ESM.jpg]
